# A Production and Fractionation Protocol for Polyvinyl Chloride Microplastics

**DOI:** 10.3390/mps6010015

**Published:** 2023-01-31

**Authors:** Siebe Lievens, Evelynn Vervoort, Giulia Poma, Adrian Covaci, Mik Van Der Borght

**Affiliations:** 1 Research Group for Insect Production and Processing, Faculty of Engineering Technology, Department of Microbial and Molecular Systems (M2S), KU Leuven Campus Geel, Kleinhoefstraat 4, 2440 Geel, Belgium; 2 Toxicological Centre, Faculty of Pharmaceutical, Biomedical and Veterinary Sciences, University of Antwerp, Campus Drie Eiken, Universiteitsplein 1, 2610 Antwerp, Belgium

**Keywords:** microplastics, cryogenic grinding, separation, wet sieving, PVC

## Abstract

Concerns about the presence of microplastics in the environment has increased in recent years, prompting more attention from scientists. Thorough exposure studies using artificially produced microplastics containing additives are required to assess their potentially hazardous effects. Therefore, an efficient microplastic production and fractionation protocol was established using a cryogenic grinding and wet-sieving approach. The developed cryogenic grinding method was able to produce (20–40 g/h) polyvinyl chloride (PVC) microplastics having a volume-weighted mean particle size of 391 µm and a span of 2.12. Performing a second grinding cycle on the same particles resulted in microplastics which were smaller (volume-weighted mean size = 219 μm) and had a narrower particle size distribution (span = 1.59). In addition, the microplastics were also fractionated into different particle size ranges using a vibrating wet-sieving tower. The latter technique allowed separating 10 g of PVC microplastics into seven different fractions using six sieves (Ø 200 mm) for 30 min while shaking. By using the developed method, PVC microplastics could easily be made and fractionated into desired particle-size ranges. The proposed protocol could also be adjusted to produce and fractionate microplastics of other plastics.

## 1. Introduction

Microplastics are defined as plastic particles with dimensions between 1 µm and 5 mm and can be subdivided into small (1 µm–1 mm) and large (1 mm–5 mm) microplastics [1,2,3]. Currently, these particles are found globally in the environment, leading scientists to research their impact on humans and the environment [2,3]. Traces of microplastics are also detected in different (wrapped) foods (e.g., fruit, vegetables and seafood) [1,4,5], whereby the exposure of humans cannot be avoided. Furthermore, microplastics are also linked to several potential health issues (e.g., oxidative stress, carcinogenicity, etc.) [3]. These health issues can be induced in two ways: physically by the plastics themselves (e.g., blocking the gastrointestinal tract), and chemically by monomers (e.g., vinyl chloride), degradation products of the polymers, or chemical additives (e.g., phthalates) present in the plastics [6,7]. To date, several studies have been performed to quantify microplastics to get insights into the potential risk linked to the amount present [8], but exposure studies, considering the plastic additives, are still limited [7].

To gain knowledge of how and to what extent microplastics can affect certain organisms, animals, and humans, thorough exposure studies are required. Possible effects can be studied using artificially produced microplastics containing additives as used in various applications. To date, a few methods to produce microplastics are described in the literature. For example, Cole (2016) developed a method using a cryogenic microtome, which is less suitable for producing larger amounts of microplastics [9]. Moreover, cryogenic milling is a technique to produce microplastics of various sizes from commercial plastics, such as low-density polyethylene, polypropylene, polyamides, polyurethanes and polyvinyl chloride (PVC) [10,11,12,13]. However, the procedures are not described in detail for PVC foil, and the high plasticizer content in PVC foil (making it less brittle) challenges the grinding procedure [14]. Additionally, a commonly used method to fractionate materials based on their particle size is a sieving procedure. This technique is also mentioned for microplastics, but its use is still limited [10,11,12]. The latter results from the fact that dry microplastic particles aggregate due to electrostatic charges, resulting in incomplete fractionation. Instead, by using water (wet sieving), that phenomenon can be reduced, minimizing agglomeration [15]. As a result, such a method can be used for more extensive exposure studies, which require higher amounts of well-defined microplastics having a narrow particle-size distribution. However, a detailed approach is lacking. Therefore, this study aimed to develop a standardized protocol to produce small PVC microplastics in considerable quantities and fractionate them into different particle size ranges. Since PVC foil is particularly difficult to mill into microplastics due to its high plasticizer content, this procedure can easily be extended to other polymer types (e.g., polyethylene, polypropylene, and polyethylene terephthalate). As a result, researchers can thus perform larger exposure studies, which requires higher amounts of well-defined microplastics with a narrow particle size distribution.

## 2. Experimental Design

**Figure 1:**
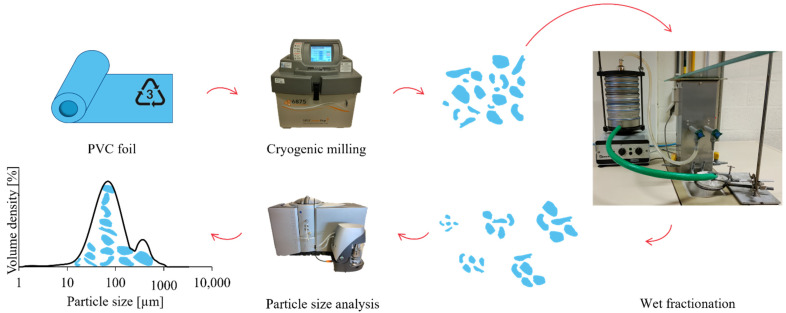
General workflow for the microplastic production and fractionation and the determination of their particle-size distribution.

A microplastic production method using a cryogenic grinder was developed to produce PVC microplastics in considerable volumes (20 to 40 g/h). After production, the PVC microplastics were separated into different fractions having smaller particle-size distributions using a vibrating wet-sieve tower. The latter contained six sieves (Ø 200 mm) having different mesh sizes ranging from 75 to 500 μm. In the last step, the microplastic fractions were analysed for their particle size through a particle-size analyser, based on laser diffraction. A general overview of the different steps is presented in Figure 1, which will be explained in detail in the Procedure section.

### 2.1. Materials

- Polyvinyl chloride foil (Flexfilm, Schoonhoven, The Netherlands);
- Scissors;
- Cryo-Gloves (Tempshield, Mount Desert, Maine, USA);
- Cryo-Apron protective coat (Tempshield, Mount Desert, Maine, USA);
- Safety goggles;
- Headband earmuff with uniform attenuation (Honeywell Safety Products, San Diego, California, USA, Cat. No.: 1011142);
- Liquid nitrogen (Air Liquide, Paris, France);
- Large grinding vials (Instrument Solutions, Nieuwegein, The Netherlands, Cat. No.: ES05-6801);
- Large vial extractor (Instrument Solutions, Nieuwegein, The Netherlands, Cat. No.: ES05-6808);
- Glass vessel with metal lid;
- Retsch stainless steel woven wire mesh sieves—Ø 200 mm (75 μm, 106 μm, 150 μm, 250 μm, 355 μm, and 500 μm mesh sizes) (Verder NV, Aartselaar, Belgium, Cat. no.: 60.131.000075, 60.131.000106, 60.131.000150, 60.131.000250, 60.131.000355, 60.131.000500);
- Retsch venting rings—Ø 200 mm (Verder NV, Aartselaar, Belgium, Cat. no.: 69.221.0025);
- Retsch collecting pan with outlet—Ø 200 mm (Verder NV, Aartselaar, Belgium, Cat. no.: 69.420.0050);
- Retsch universal wet-sieving lid (Verder NV, Aartselaar, Belgium, Cat. no.: 32.481.0015);
- Silicone rubber tubing, 10 mm inner diameter (Deutsch & Neumann, Hennigsdorf, Germany, Cat. no.: 273460040);
- PVC tubing, 19 mm inner diameter (GARDENA, Ulm, Germany, Cat. no.: 18022-20);
- Stainless steel worm-drive tube clamps for clamping range 16–27 mm (NORMA, Maintal, Germany, Cat. no.: 100024003);
- Filter paper 4.0–12.0 μm with ca. 0.1% ash content (Macherey–Nagel, Düren, Germany, Cat. no.: 90600201);
- Zincked steel stand (BOCHEM, Weilburg, Germany, Cat. no.: 310505151, 310505002);
- Aluminium boss heads (BOCHEM, Weilburg, Germany, Cat. no.: 310505332);
- Steel universal clamp (BOCHEM, Weilburg, Germany, Cat. no.: 310505425);
- Steel support ring (BOCHEM, Weilburg, Germany, Cat. no.: 310505502);
- 50 mL beaker (DURAN^®^, Darmstadt, Germany, Cat. no.: 112110617).

### 2.2. Equipment

- Freezer/Mill 6875^®^ (Instrument Solutions, Nieuwegein, The Netherlands, Cat. No.: ES05-6875A-230);
- Retsch vibrating sieve system Vibro (Verder NV, Aartselaar, Belgium);
- Universal drying oven (Memmert, Schwabach, Germany, Cat. no.: UFB 500);
- Mastersizer^®^ 3000 with Hydro EV^®^ (Malvern Panalytical, Worcestershire, UK).

## 3. Procedure

The protocol for producing and fractionating PVC microplastics and determining their particle size distribution is divided into three parts. These procedures are described in detail in the following subsections.

### 3.1. Microplastic Production (40 min)

1. Fill the cryogenic grinder with liquid nitrogen to the mark and close the lid slowly (to prevent splashing) to fully cool the system. Wear cryogenic protection (gloves, apron, and safety goggles) to be protected from possible liquid nitrogen splashes;
2. Cut the appropriate plastic (PVC) into pieces (1 × 1 cm) using scissors;
3. Fill a large (100 mL) grinding vial half with the appropriate plastic pieces, which was 15 to 20 g of PVC in this case;
4. Place the vial(s) in the cryogenic grinder and close the lid slowly;
5. After precooling the vial(s) by submerging them in liquid nitrogen for 5 min, run the grinding procedure consisting of eight cycles of 2 min grinding and 2 min cooling, with a grinding rate of 12 cps (cycles per second). Wear earmuffs during grinding;
6. Open the lid and take out the vial(s);
7. Open the vial(s) using the vial extractor. 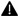 Caution, the plugs may fly off due to pressure changes resulting from temperature differences;
8. Transfer the microplastics into a glass vessel.

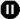
**PAUSE STEP** The microplastics can be stored in the glass vessel pending fractionation, particle-size analysis or other experiments.

### 3.2. Microplastic Fractionation (100 min)

**Figure 2:**
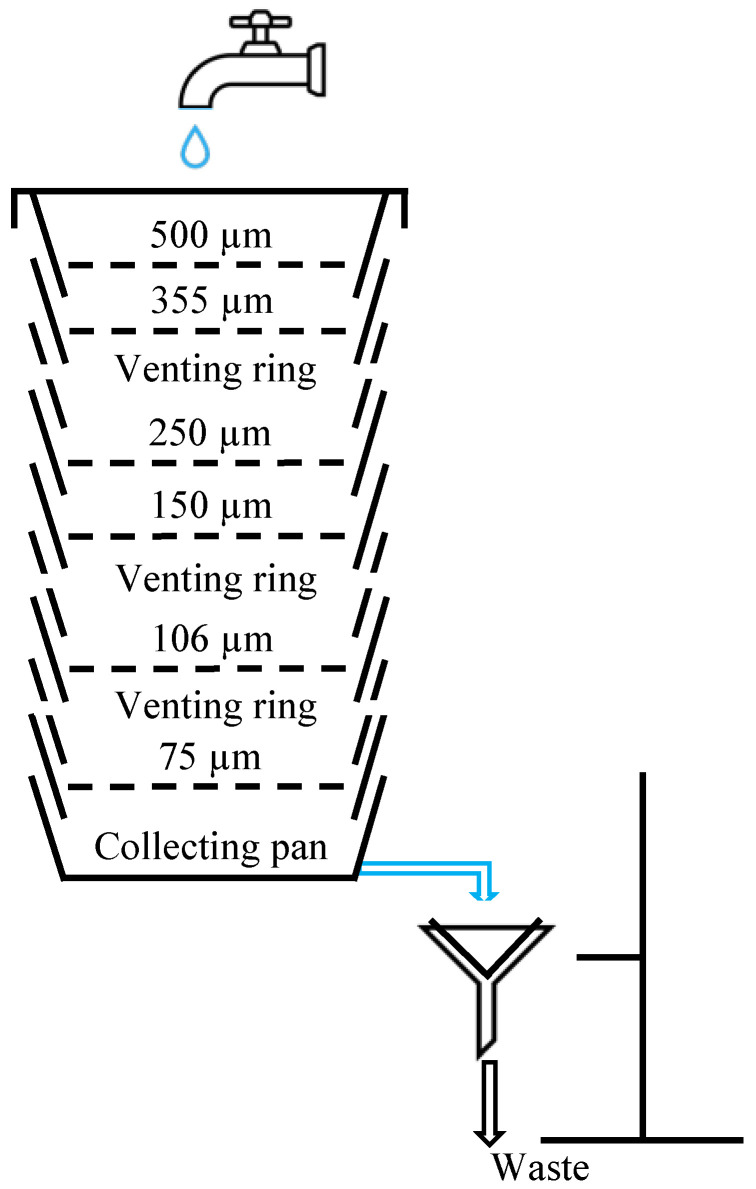
Schematic overview of the fractionation setup.

1. Build the sieve setup as schematically shown in Figure 2. The sieve tower consists of a sieve shaker with accompanying sieves, venting rings, wet-sieving lid and collecting pan. Connect the lid to the tap with a tube (Ø 10 mm) and the outlet of the collecting pan to another tube (Ø 19 mm), and secure those tubes with worm-drive tube clamps. The outlet tube drains into the sink, but a filter is placed in between, preventing the smallest microplastics from entering the drain. A stand with boss heads, a support ring, a clamp, and a funnel is also used to keep the tube and the filter in place.
2. Suspend the microplastics (10 g) into a beaker containing tap water (50 mL);
3. Pre-wet the sieves using tap water and check for clogging;
4. Transfer the suspended microplastics onto the sieve tower and rinse the beaker five times with (tap) water;
5. Place the sieving lid with the water nozzle on the sieving tower;
6. Turn on the water supply (tap water) and wash the sieve tower while shaking (80 Hz) for 30 min;
7. Turn off the water supply and sieve shaker;
8. Transfer the microplastics from each sieve onto a paper filter and dry all fractions using a drying oven at 105 °C for 2 h.

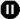
**PAUSE STEP** Collect all microplastics into different glass vessels preceding their particle-size analysis or other experiments.

### 3.3. Particle-Size Determination (20 min)

1. Open the Mastersizer 3000 software, select manual measurement and fill in the correct measurement settings:
  - Particle type: non-spherical;
  - Material: PVC (refractive index: 1.539);
  - Absorption index: 0.01;
  - Dispersant: water (refractive index dispersant: 1.33);
  - Background and sample measurement time: 10 s;
  - Number of measurements: 3;
  - Analysis model: general purpose.
2. Initialize the Mastersizer 3000 and measure background before starting the analysis;
3. Suspend the microplastics into the analysis beaker (600 mL) of the Mastersizer 3000 containing 500 mL of demineralized water, while stirring at 3500 rpm until an obscuration of 5–8% is obtained;
4. Perform the particle size analyses in accordance with ISO 13320 while stirring the suspension at 3100 rpm. The stirring rate must be sufficiently high to maintain a stable dispersion but not too high, to prevent the formation of air bubbles, which cause incorrect measurements.

The Mastersizer 3000 software expresses the particle size distribution by giving the volume-weighted percentile values *Dv*(10), *Dv*(50) and *Dv*(90). These values represent the size (in µm) below which 10%, 50%, and 90% of all particles are found, respectively. Another characteristic value of the sample is the volume-weighted mean particle size, calculated using Equation (1) according to Čurlin et al. (2021) [16].

(1) $$\mathrm{Volume}-\mathrm{weighted}\;\mathrm{mean}\;\mathrm{particle}\;\mathrm{size}=\;\frac{\sum v\left(i\right)\;d\left(i\right)}{\sum v\left(i\right)}$$

where *v*(i) represents the volume of particles of size *d*(i) (µm). Further, the width of the particle-size distribution, also called the span, is calculated using Equation (2), according to Čurlin et al. (2021) [16].

(2) $$\mathrm{Span}=\;\frac{Dv(90)\;-\;Dv(10)}{Dv(50)}$$

## 4. Expected Results

**Figure 3:**
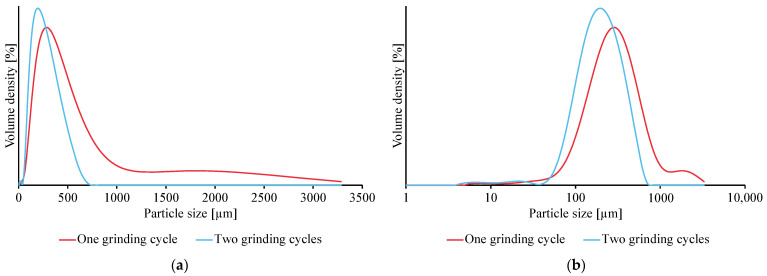
Particle-size distribution of PVC microplastic batches after performing one and two cryogenic grinding cycles. The particle size distributions are depicted on a (**a**) linear and (**b**) logarithmic particle size scale.

Small PVC microplastics were successfully produced using the described method. By performing one grinding cycle (5 min precooling and eight grindings for 2 min with 2 min inter-cooling at a grinding rate of 12 cps), PVC microplastics with a volume-weighted mean particle size of 391 ± 5 µm were made. This batch of microplastics showed a broad particle-size distribution having a span of 2.12 ± 0.03 (Table 1), which could be narrowed by further grinding the plastics with an additional grinding cycle (in total: 5 min precooling and 16 grindings for 2 min with 2 min inter-cooling at a grinding rate of 12 cps). By applying two grinding cycles, the weighted mean particle size dropped to 219 ± 3 µm, and the distribution was narrowed considerably to a span of 1.59 ± 0.03 (Figure 3). Moreover, it was found that the tail of the particle size distribution, corresponding to microplastics of a considerably larger size, was eliminated, meaning that the larger particles were all ground into smaller ones (Figure 3a,b).

**Figure 4:**
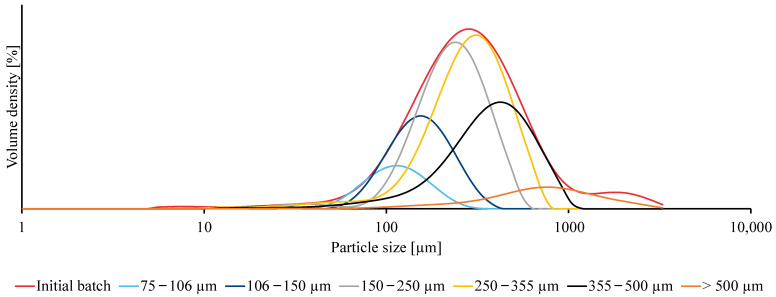
Particle size distributions of the initial PVC microplastics batch (one grinding cycle) and its fractions after wet sieving 10 g microplastics for 30 min while shaking.

Performing the grinding cycle once resulted in microplastics having a broad particle-size distribution. These microplastics could be used to conduct specific research with microplastics, having the advantage of being a realistic situation. However, a smaller and more specific particle-size range could be used for purpose-oriented research. For the latter, a wet-sieving procedure could fractionate the initial batch of microplastics into several fractions, each with a smaller span. Therefore, the initial batch of microplastics was transferred to the upper sieve, having the biggest mesh size (500 µm), of the sieve tower. In addition to the choice of sieve mesh sizes, the sieve area and amount of microplastics added to the sieve tower played a crucial role in sieve clogging. In this case, 10 g of microplastics was added as an optimal amount for the Ø 200 mm sieves. Adding a lower amount did not considerably improve the particle fractionation, making the procedure less efficient, whereas a higher amount caused clogging and, consequently, incomplete fractionation. Moreover, the 500 µm and 355 µm sieves were also added to the sieve tower to prevent blockages of the underlying sieves with smaller mesh sizes. Along with adding an optimal amount of microplastics, applying an optimal sieving time is also required, which was, in this case, 30 min for 10 g of PVC microplastics (Figure 4). Using a shorter sieving time resulted in an incomplete separation (i.e., smaller particles in the bigger fractions) while applying a longer sieving time did not lead to a significantly improved fractionation.

The wet sieving procedure provided seven fractions of microplastics. However, the yield of the fraction with presumably the smallest particles (<75 µm) was too low to perform particle-size measurements. The remaining fractions had increasing particle sizes and narrower spans than the initial batch. The span decreased from 2.12 for the initial batch to less than 1.82 for its fractions. As the differences between the mesh sizes of consecutive sieves became larger with increasing mesh size, so did the span of each fraction, which can be observed in Table 2. Additionally, the wider span of the bigger particle-size fractions (i.e., 355–500 µm and >500 µm) may be due to the hydrophobicity of the particles [17]. As a result, clusters are being formed, allowing smaller particles to be encapsulated by bigger ones and preventing them from going through the mesh of the used sieve. Furthermore, for each fraction, the mesh sizes of the lower and upper sieves did not always correspond well to the size of the smallest and largest particles (depicted as the *Dv*(90) and *Dv* (10) values) in each of those fractions. The latter can be explained by the fact that sieves are given a nominal mesh size, which might deviate from their actual size. Moreover, Lievens et al. (2022) proved that microplastics produced using a cryogenic approach are irregularly shaped [18]. Hence, the particle-size analysis based on laser diffraction assumes spherical particles in its optical model. For that reason, the resulting particle sizes differ between laser diffraction and sieving [19].

This study used PVC foil as a model plastic to develop a protocol for producing and fractionating microplastics with mean particle sizes from 120 mm to 886 mm. However, this procedure can easily be adapted for other plastic types. A first trial was executed for polyethylene (PE), polypropylene (PP), polystyrene (PS), and polyethylene terephthalate (PET) using the same procedure as for PVC. This trial resulted in microplastics having volume-weighted mean particle sizes of 685, 462, 645 and 439 µm for PE, PP, PS, and PET, respectively. The obtained particle sizes are higher compared to PVC, showing that the physical properties of the source material (e.g., brittleness, thickness of initial material, additive composition, etc.) have a significant impact on the obtained particle size. Therefore, for each individual plastic source the procedure needs to be optimised. Further, the fractionation process could be ameliorated by using a surfactant solution to improve the wetting of hydrophobic plastic types. If a surfactant is used, a reservoir and a suitable pump to circulate this solution should be included in the experimental setup.

## 5. Conclusions

A protocol to produce PVC microplastics in substantial quantities (20 to 40 g/h) and subsequently to separate them into different fractions of distinct particle sizes having narrow spans was successfully developed. First, the PVC microplastics were easily produced using a cryogenic grinder filled with 15 to 20 g of PVC foil, precooled for 5 min and, depending on the desired particle size, ground eight or 16 times for 2 min with 2 min of inter-cooling at a grinding rate of 12 cycles per second. As a result, PVC microplastics were obtained having a volume-weighted mean particle size of 391 ± 5 µm with a span of 2.12 ± 0.03 (for eight grindings). The volume-weighted mean particle size decreased to 219 ± 3 µm and the span to 1.59 ± 0.03 when a second grinding cycle was applied. Next, 10 g of these microplastics were fractionated, using a vibrating wet-sieving tower equipped with six sieves (Ø 200 mm) having the desired mesh sizes, resulting in seven microplastic fractions. The wet sieving procedure narrowed the span from 2.12 to lower than 1.82 for each PVC microplastic fraction. Finally, the obtained microplastic (fractions) could be effectively used for multiple purpose-oriented studies.

## Figures and Tables

**Table 1:** Volume-weighted percentile values (*Dv*(*x*)), volume-weighted mean sizes (each in µm) and spans of the microplastics obtained after performing one and two grinding cycles. Results are depicted as the average of three replicates ± the standard deviation.

	One Grinding Cycle	Two Grinding Cycles
*Dv*(10)	111 ± 1	88.2 ± 0.9
*Dv*(50)	279 ± 1	192 ± 2
*Dv*(90)	703 ± 12	394 ± 6
Volume-weighted mean size	391 ± 5	219 ± 3
Span	2.12 ± 0.03	1.59 ± 0.03

**Table 2:** Differences between mesh sizes of the upper and lower sieve (in μm), percentile (*Dv*(*x*)) values (in μm), volume-weighted mean sizes (in μm) and span for the PVC microplastic fractions after wet sieving 10 g microplastics for 30 min. Each fractionation was performed in triplicate. The table shows average values of those three replicates ± the standard deviation.

Mesh Sizes of the Lower and Upper Sieve	75–106	106–150	150–250	250–355	355–500	>500
Δ(mesh size sieves)	31	44	100	105	145	-
*Dv*(10)	64.8 ± 1.2	87.2 ± 0.1	121 ± 4	156 ± 27	185 ± 49	279 ± 41
*Dv*(50)	112 ± 2	152 ± 1	224 ± 7	304 ± 29	405 ± 50	762 ± 106
*Dv*(90)	188 ± 7	253 ± 4	380 ± 10	519 ± 30	718 ± 47	1673 ± 315
Volume-weighted mean size	120 ± 3	161 ± 2	238 ± 7	321 ± 28	430 ± 47	886 ± 131
Span	1.10 ± 0.06	1.10 ± 0.02	1.15 ± 0.01	1.20 ± 0.10	1.33 ± 0.16	1.82 ± 0.26

## Data Availability

Original data are available on request.
